# Experimental Analysis of the Machinability of 94 WC–6 Co by Die-Sinking EDM

**DOI:** 10.3390/ma17246032

**Published:** 2024-12-10

**Authors:** Unai Salvide-González, Ignacio Puertas-Arbizu, Carmelo Javier Luis-Pérez

**Affiliations:** 1 Engineering Department, Public University of Navarre, Campus de Arrosadía s/n, 31006 Pamplona, Spain; salvide.133623@e.unavarra.es; 2 Institute for Advanced Materials and Mathematics (INAMAT2), Public University of Navarre, Campus de Arrosadía s/n, 31006 Pamplona, Spain

**Keywords:** EDM, WC, surface roughness, wear, MMR, DOE

## Abstract

Cobalt-bonded tungsten carbide (WC-Co) is widely used in heavy-duty machining applications due to its exceptional hardness and wear resistance, and it is increasingly being adopted in industries such as aerospace and the automotive sector, among others. Its superior mechanical properties make it difficult to machine with conventional methods such as turning or milling. Electrical Discharge Machining (EDM) has emerged as an efficient alternative, as it allows for the machining of hard materials to be carried out without direct contact between the tool and the workpiece, provided that the material has sufficient electrical conductivity. In this study, a multilevel Design of Experiments (DOE) was conducted to analyze the influence of EDM parameters—specifically, the current intensity and pulse time—on the surface roughness (SR), electrode wear (EW), and material removal rate (MRR) for 94WC–6Co. The results indicate that the current intensity was the most significant factor across all responses, while the pulse time played a secondary role. Surface finishes as low as Ra = 0.47 μm were achieved at I = 2 A and t_i_ = 10 μs. For each outcome variable, mathematical models were obtained in order to improve the EDM processes and better understand the machining of WC-Co.

## 1. Introduction

Tungsten carbide, commonly referred to as hard metal, is a conductive ceramic highly valued in industry for its outstanding mechanical properties, particularly its remarkable hardness and resistance to abrasion and wear [1,2]. Its metallic appearance and superior performance compared to high-grade steels have positioned tungsten carbide at the forefront of fields such as cutting and machining technology, and it is increasingly being adopted in industries such as aerospace, the automotive sector, mining, and nanomaterials [3,4,5], as well as in medical fields [6], among others.

However, its outstanding properties also make tungsten carbide extremely difficult to machine using conventional processes such as turning or milling. As a result, Electrical Discharge Machining (EDM) has emerged in recent years as an efficient and precise alternative for machining this material. The primary advantage of EDM is that it does not require direct contact between the tool and the workpiece, thus making it capable of machining very hard materials without direct contact, provided they have sufficient electrical conductivity to generate a spark. Key machine variables such as the current (I), open-circuit voltage (U), and duty cycle (η), defined by pulse-on (t_i_) and pulse-off (t_o_) times, are particularly important to examine. Additionally, factors such as the tool material, dielectric fluid flushing pressure, and the type of machine head movement may also be considered, as they can significantly impact the machining outcomes. While the behavior of EDM is well understood for traditional materials such as steel, with established process parameters, this is not the case for conductive ceramics. In recent years, there has been a collective effort to study how this material responds to various EDM variables in order to optimize the industrial manufacturing process. These efforts focus on analyzing the parameters and machining elements that may lead to the achievement of, for instance, high-quality surface finishes or high machining speeds for competitive industrial production.

Several studies have examined key process responses, such as the surface quality, machining time, and tool wear, for various advanced ceramics. Lee and Li [7] conducted a comprehensive study on the EDM machining of tungsten carbide (WC), which analyzed the effects of the electrical parameters, electrode materials, and polarity. They confirmed that it is difficult to prioritize all three critical responses—the material removal rate (MRR), electrode wear (EW), and arithmetic mean of the roughness profile (Ra)—simultaneously, as improvements in one typically come at the cost of the others. Thus, specific strategies must be developed based on the desired outcome. They found out that, for intervals including a pulse time of 12.8–200 μs, a peak current of 16–64 A, and an open-circuit voltage of 80–200 V, the optimal conditions for the wear and surface roughness happened at a pulse time of 12.8 μs, a peak current of 24 A, and an open-circuit voltage of 120 V by using a CuW electrode. For a better surface quality and machining speed, a reversed circuit polarity is recommended, a result which is also supported by Jahan et al. [8] in their review of EDM’ed WC, which noted that more energetic processes caused rougher surfaces and increased cracking.

Işık et al. [9] varied the levels of the pulse time to 25–100 μs, the pause time to 15–60 μs, and the peak current to 6–25 A, and they achieved an increase of 23.95% on the EDM process performance indicators for the machining of WC–15Co by employing a Taguchi–Gray rotational analysis. The optimal conditions were found at a pulse time of 25 μs, a pulse interval of 100 μs, and an intensity of 6 A. In their study, they discovered that the current intensity was the most influential factor for all of the outcome parameters, where the Ra, MRR, and tool wear rate increased notably with it. On the other hand, increasing the interval time—which is equivalent to decreasing the duty cycle—was found to have a minimizing effect on all of the parameters and allowed for better debris removal to be obtained on the processed surface.

Another statistical study was conducted by Assarzadeh and Ghoreishi [10], who employed copper electrodes on a 94WC–6Co sample using a rotatable central composite design. Their experimental procedure involved varying the levels of current (1–5 A), the pulse-on time (25–125 μs), the duty cycle (40–80%), and the voltage (40–80 V) in order to assess the material responses. Additionally, a linear optimization approach was used to identify the optimal parameters for various machining strategies. The results revealed that the discharge current and duty cycle had a significant impact on the MRR, with increases in both parameters leading to a higher MRR due to the elevated discharging energy. The tool wear rate was the most influenced by the current, pulse-on time, and duty cycle, with it being reduced by employing lower current levels and longer pulse times. The surface roughness (Ra) was predominantly affected by the discharge current, pulse time, and duty cycle, with smoother surfaces obtained through shorter pulse times and relatively higher discharge currents, thus producing smaller and shallower craters. Similarly, Mohd et al. [11] employed the Taguchi method using a graphite electrode on a WC sample in order to optimize the EDM process for tungsten carbide. Their study aimed to investigate the impact of various EDM parameters—namely, the peak current, voltage, pulse time, and interval time—on the machining efficiency and surface quality. The results demonstrated that the peak current significantly affected both the electrode wear rate and the surface roughness, while the pulse time had the most pronounced effect on the MRR. Additionally, scanning electron microscopy (SEM) revealed that higher energy processes, particularly those involving a higher voltage, resulted in an increased crater size on the machined surface.

A typical application of EDM is in mold production, which often involves machining complex geometries, such as drilling holes and microholes. Several studies have focused on analyzing the EDM drilling process in advanced ceramics. For instance, Torres-Salcedo et al. [12] investigated the statistical influence of the previously mentioned electrical parameters along with several electrode materials (C, Cu, CuC, and CW) and different levels of electrode rotational speeds (0–40 rpm) during the drilling of SiSiC. They examined the MRR, EW, Ra, and the structural quality of hole entrances, and they identified the electrode material, current intensity, and pulse time as the most influential parameters, whereas the rotational speed was not particularly impactful. Optimal materials were identified for each machining strategy. For instance, to achieve the fastest MRR, a CuW electrode tool was recommended along with a high-current roughing regime. Similarly, Kliueve et al. [13] conducted a study on SiC and SiSiC samples, and they also determined the best working strategies to achieve a favorable MRR and EW for both materials, which were met at a high current of 24 A and a low pulse time of 8 μs. They also observed a phenomenon where the electrical conductivity of SiSiC increased due to copper deposition from the electrode onto the workpiece, an idea that has been reported to facilitate machining in less conductive ceramics [14]. The development of these sorts of innovative techniques is becoming increasingly popular, as they allow for the further improvement of process outcomes. For instance, Gattu and Yan [15] tried mixing carbon nanofibers (CnFs), semiconductive silicon (Si), and alumina (Al_2_O_3_) powders with traditional mineral oil in concentrations from 0 to 1 g/L in order to search for useful interactions. Their analysis concluded that CnFs and Al_2_O_3_ achieved smaller craters on the machined WC surface, and that, under the right voltage, the use of either powder showed a notable increase in the MRR.

This research aims to deepen the understanding of the effect of two of the most relevant EDM electrical parameters (the current intensity and pulse time) on the main EDM outcomes—the surface roughness, material removal rate, and tool wear—for advanced ceramics such as WC-Co. The resulting models, combined with the findings of the chemical characteristics of the machined surfaces, provide valuable insights into the process.

## 2. Materials and Methods

**Figure 1:**
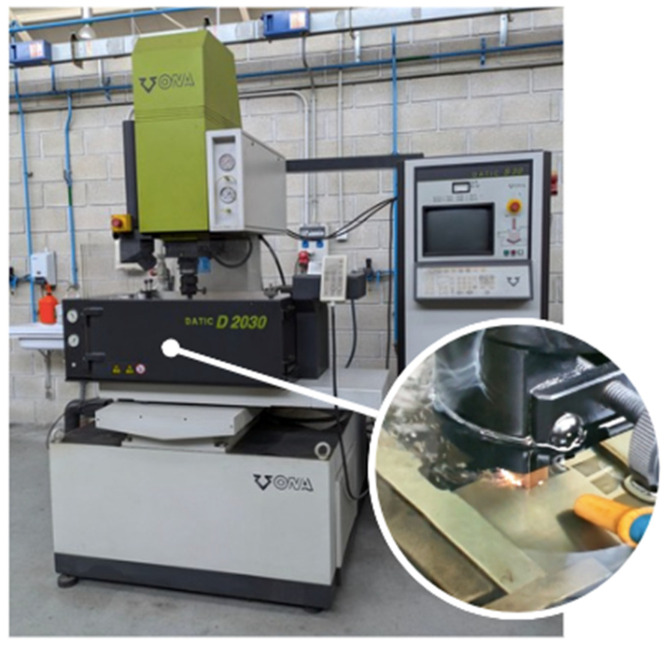
ONA DATIC D-2030-S die-sinking machine.

The experiments were conducted on 50 mm × 50 mm × 4 mm samples of cobalt-bonded tungsten carbide using a conventional die-sinking EDM machine, the ONA DATIC D-2030-S (ONA Electroerosión S.A., Durango, Spain), as depicted in Figure 1. The samples were secured and squared using a mechanical clamp, which was then attached to the worktable via a magnetic hold.

The conductive ceramic sample consisted of 94% tungsten carbide, with the remaining 6% comprising a cobalt metallic matrix. The selected grade of the WC-Co has a thermal conductivity of 100 W/mK and an electrical resistivity value of 2·10^−6^ Ωcm. Tungsten carbide is a conductive ceramic known for its exceptional hardness and wear resistance, where this allows for its use in harsh environments and at high working temperatures from 600 °C up to 1200 °C at local points [16]. Its electrical properties also make it suitable for machining via electrical discharge, thus yielding good results. The cobalt content plays a major role in the process, since it may vary the surface roughness parameters significantly due to the metallic nature of cobalt [17]. The studied samples do not contain any additional alloying elements, but this technique has been reported to notably influence the EDM process of WC-Co. Bonny et al. [18] investigated the influence of the cobalt (Co) content and the addition of grain-growth inhibitors, such as chromium (Cr) and vanadium (V), on the surface integrity and MRR. Their findings demonstrated that both factors significantly impacted these outcomes due to their effect on the thermal conductivity of the samples. Specifically, a lower Co content resulted in rougher surfaces overall. Additionally, for WC-10Co, the introduction of grain-growth inhibitors led to a 12.96% improvement in the MRR.

The machine head was equipped with prismatic electrolytic copper electrodes, which had a rectangular cross-section of 12 mm × 8 mm. Based on this geometry and the EDM-recommended maximum current density of 7.75 A/cm^2^, an upper current level of 6 A was chosen for the experiment, which is below the limit value for the selected electrode section. It has been reported that electrode geometry significantly affects several outcome parameters in EDM processes. For example, when machining steels, circular-section electrodes are generally considered the most effective option. Among non-circular electrodes, square geometries are recommended due to their lower wear rates and the superior surface finish they produce [19,20]. Prior to each experiment, both surfaces underwent thorough cleaning. For the electrode, a milling process was performed to achieve a smooth, mirror-like finish, so that each experiment would begin with an electrode of precise dimensions. Several studies have described the changes in the morphology of copper electrodes due to wear. For instance, Khan [21] examined the wear on the horizontal and vertical axes, which occurred mainly at the lower edges of the employed 15 mm × 15 mm square copper electrodes. He discovered that the cross-sectional area experienced greater wear than the material along the longitudinal axis. In contrast, Ramabalan et al. [22] studied electrodes with side dimensions ranging from 0.5 mm to 0.8 mm, and they observed the opposite effect due to the horizontal dimension being significantly smaller than the vertical one. They also identified that the current intensity was the most influential parameter in the wear, and, for this range of dimensions, a larger cross-sectional area was associated with increased vertical wear. In this case, the electrode tip developed a clear hemispherical shape. This wear process, which initiates at the tips and edges of the electrodes, is explained by the skin and tip effects, which lead to charge accumulation in these regions before the discharge. Furthermore, it has been demonstrated that polarity determines whether wear develops the electrode tip into concave or convex shapes [23].

**Figure 2:**
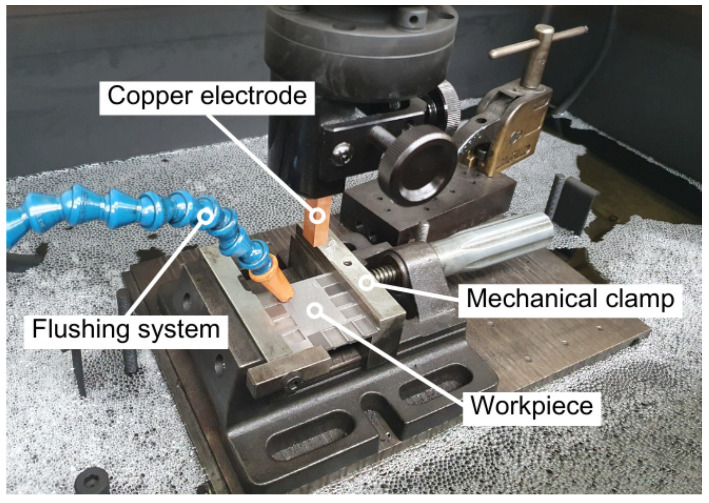
Arrangement of the worktable elements.

To ensure effective particle removal and maintain the efficiency of the erosion process, a side jet system with a constant pressure of 30 kPa was employed for external flushing. The use of a proper dielectric flushing system is key, as it allows for the formation of surface cracks to be reduced due to localized overheating in low thermally conductive materials such as WC-Co [24]. An IME-Series Oelheld mineral oil was chosen as the dielectric fluid due to its widespread use and ability to improve the process stability in die-sinking applications. Figure 2 illustrates the full configuration of the worktable elements arranged for one of the experiments.

The CNC machine was configured to perform simple vertical movements on the sample for each experiment. The head was programmed to automatically stop either upon reaching a mark depth of 1 mm or after 2 h of operation. Subsequently, the three response variables were measured. The MRR and EW were defined by Equations (1) and (2), respectively, as follows:

(1) $$MRR=\frac{\mathrm{Volume}\;\mathrm{of}\;\mathrm{workpiece}\;\mathrm{material}\;\mathrm{removed}}{\mathrm{Time}\;\mathrm{of}\;\mathrm{machining}}$$

(2) $$EW\;\left(\%\right)=\frac{\mathrm{Volume}\;\mathrm{of}\;\mathrm{electrode}\;\mathrm{material}\;\mathrm{removed}}{\mathrm{Volume}\;\mathrm{of}\;\mathrm{workpiece}\;\mathrm{material}\;\mathrm{removed}}\;100$$

The volume differences for the tungsten carbide samples and the copper electrodes were determined by measuring their masses at the beginning and end of each experiment. These mass measurements were then converted to volumes using density values of 14.95 g/cm^3^ for tungsten carbide and 8.96 g/cm^3^ for copper. A Mettler Toledo XS 104 scale (Mettler Toledo, Columbus, OH, USA) was used for these measurements, which has a precision of (0.04 mg + 2·10^−7^ × weight).

For measuring the SR, the standardized parameters of the arithmetical mean deviation (Ra) and the maximum height (Rt) of the roughness profiles were used, as specified by ISO 21920-2:2023 [25]. The Ra is widely recognized and commonly used in the industry for assessing the surface roughness, while the Rt provides information regarding the maximum height of the analyzed roughness profile. Those parameters were measured using an ALPA SM-Rt-70 profile rugosimeter (ALPA Metrology, Pontoglio, Italy), taking five equidistant measurements in each mark, which were then averaged. The rugosimeter was equipped with a Gaussian filter with a 90° angle and a 5 μm radius diamond conical probe, which exerted a 0.12 mN force on the surface. The measurement was set to a cut-off value λc = 0.8 mm, which resulted in an evaluation length of 4 mm. All of the roughness parameter values were measured within a 5% precision range. Past research has shown that surface roughness measurements typically exhibit the highest relative uncertainty values among the studied outputs [26]. For cases with a maximum and minimum surface finish, according to the Ra, a detailed topographical analysis was performed using a Zeiss SEM microscope (Carl Zeiss, Oberkochen, Germany) at several magnifications and a Sensofar S-Mart confocal microscope (Sensofar Metrology, Terrassa, Spain) with a 20× magnification lens.

A multilevel DOE was employed to obtain second-order polynomial models for each of the studied process outcomes. Although the use of higher-order models, or even conventional or fuzzy artificial neural networks (ANNs), could have been appropriate in order to identify higher-order effects, the following results indicate that such effects are not expected, as the R^2^ values obtained for all cases, except for the EW, were above 98%. The two design parameters selected for the study were the current intensity and pulse time, as they are two of the most commonly used parameters and provide a comprehensive description of the electrical aspects of the process. Current intensity levels of 2, 4, and 6 A and pulse time levels of 10, 40, 70, and 100 μs were selected, which resulted in a total of 12 experiments. The remaining electrical key parameters, mainly the duty cycle and voltage, were set at 0.5 and −200 V, respectively. The polarity was set to negative, as this configuration has been proven by past literature to lead to significantly improved results. The main non-electrical variable involved in this study, the flushing system pressure, was set at 30 kPa. The study and statistical modeling were conducted using STATGRAPHICS Centurion 19^®^ software (Statgraphics Technologies, Inc., The Plains, VA, USA) by utilizing several of its analytical tools. The predictive capability of the obtained models is limited to the maximum and minimum levels of the current intensity and the pulse time studied. Within these limits, the models achieved a very accurate fit to the experimental data obtained from the studied 94WC–6Co samples, as indicated by the R^2^ values. Nevertheless, the use of the following expressions to extrapolate cases outside of the studied intervals or for different material grades is not recommended.

## 3. Results and Discussion of Results

Table 1 shows all 12 combinations of the input parameters I and t_i_ along with their corresponding experimental results.

### 3.1. Analysis of Surface Finish

#### 3.1.1. Ra

The statistical model calculated for the roughness parameter (Ra) is shown in Equation (3). In this case, the model was assessed with a R^2^ value of 98.45%, as follows:

(3) $$R_{a}=-1.38931+1.03408\cdot I+0.00412259\cdot t_{i}-0.1005\cdot I^{2}+\;0.00266667\cdot I\cdot t_{i}-0.0000568519\cdot t_{i}^{2}$$

**Figure 3:**
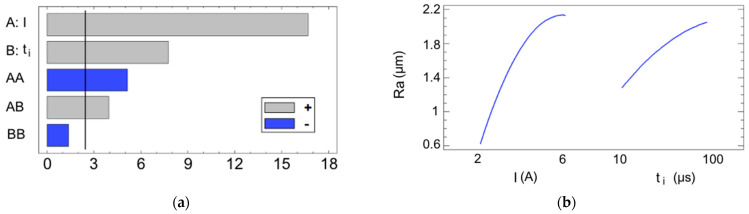
(**a**) Pareto chart for the Ra. The blue and gray colors represent the type of influence (positive or negative) that each source exerts on the magnitude of the output variable; (**b**) main effects plot for the Ra.

An analysis of variance (ANOVA) was then conducted for both of the roughness parameters to identify the most significant process variables. Variables with a *p*-value of less than 0.05, considered statistically significant, are highlighted in Table 2 for the Ra parameter. All of the sources are shown in a Pareto chart (Figure 3a), and their influence is illustrated by the main effects plot (Figure 3b).

**Figure 4:**
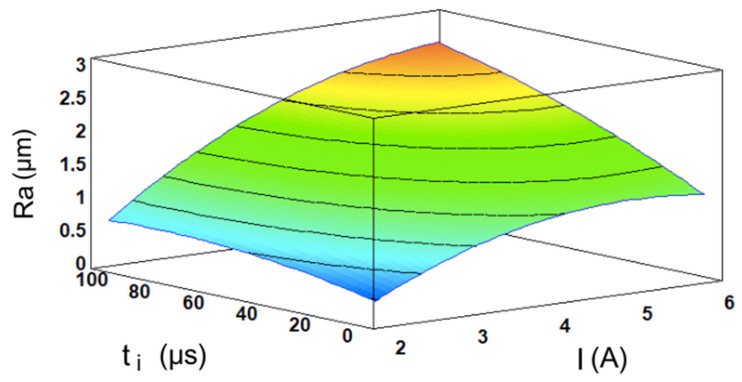
The I- and t_i_-dependent response surface for the Ra.

Based on the results, the current intensity emerges as the most influential variable, followed distantly in second place by the pulse time, the quadratic effect of the current, and the interaction effect of the current and the pulse time. Both of the main sources I and t_i_ have a positive effect on the Ra, which translates into a rougher finish. This outcome is logical, since the EDM process is based on the concept of bombarding electrons into the workpiece surface, which then release their thermic energy to create sparks up to 20,000 °C [27] that are capable of melting solid material [28]. As increasing the current levels while maintaining a constant voltage results in a greater number of electrons—or Amperes—being transferred, meaning that more energy is converted to heat, which enhances the material melting. In short, higher energy cycles create larger craters, which increase the value of the Ra. This is a typical outcome in most EDM-related studies [29,30,31,32]. Since the experiment was conducted using only two input variables, the model can be fully represented by the three-dimensional response surface defined by its equation, which is shown in Figure 4. The response surface exhibits a relatively simple geometry. Considering that higher values of the Ra correspond to poorer surface finishes, it is evident that the surface roughness deteriorates when higher values for the current intensity and pulse time are used, as justified previously. Conversely, lower levels of these variables result in improved surface finishes.

#### 3.1.2. Rt

**Figure 5:**
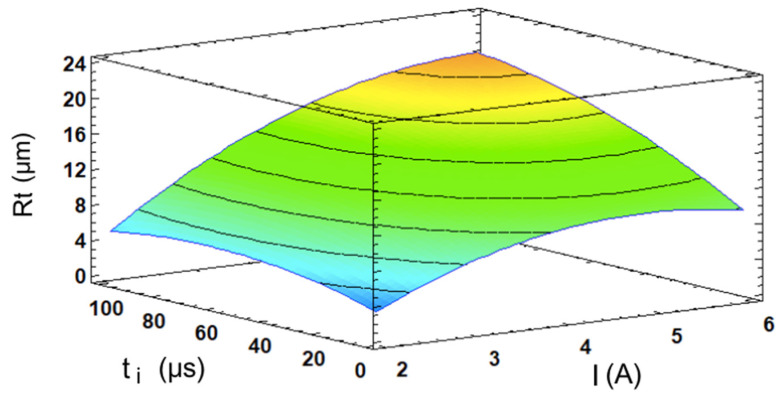
The I- and t_i_-dependent response surface for the Rt.

The model for the Rt yielded results that were very similar to the previous case regarding the statistical influence of its factors. In this instance, the model achieved an R^2^ value of 98.32%, and is defined by Equation (4) and illustrated by the response surface in Figure 5, which exhibits a similar curvature to the one shown in Figure 4 for the Ra.

(4) $$R_{t}=-10.5658+8.56038\cdot I+0.0502456\cdot t_{i}-0.872063\cdot I^{2}+\;0.0218317\cdot I\cdot t_{i}-0.000735\cdot t_{i}^{2}$$

**Figure 6:**
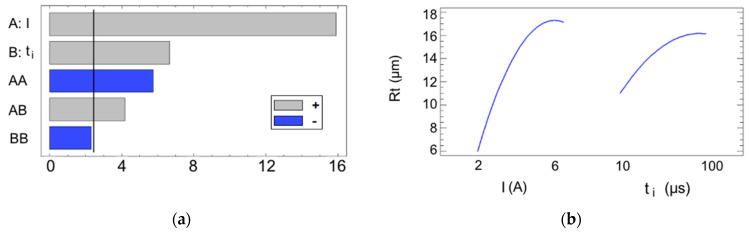
(**a**) Pareto chart for the Rt; (**b**) main effects plot for the Rt.

The analysis of variance, summarized in the Pareto chart (see Figure 6a), produced results that were similar to the Ra case in terms of the statistical significance of the factors and their ranking of importance. Once again, the current intensity proved to be the most influential factor, followed by the pulse time, the quadratic effect of the current intensity, and the interaction effect between the two factors. This is logical, since both of the parameters are inherently related; when surface irregularities increase, such as deeper valleys or higher peaks, both the average deviation (Ra) and the total height difference (Rt) will increase. A rougher surface with more pronounced features due to a higher current intensity and pulse time use will exhibit larger average deviations and a greater vertical range, thus leading to higher values for both parameters, as shown in Figure 6b.

#### 3.1.3. Surface Microscope Analysis

**Figure 7:**
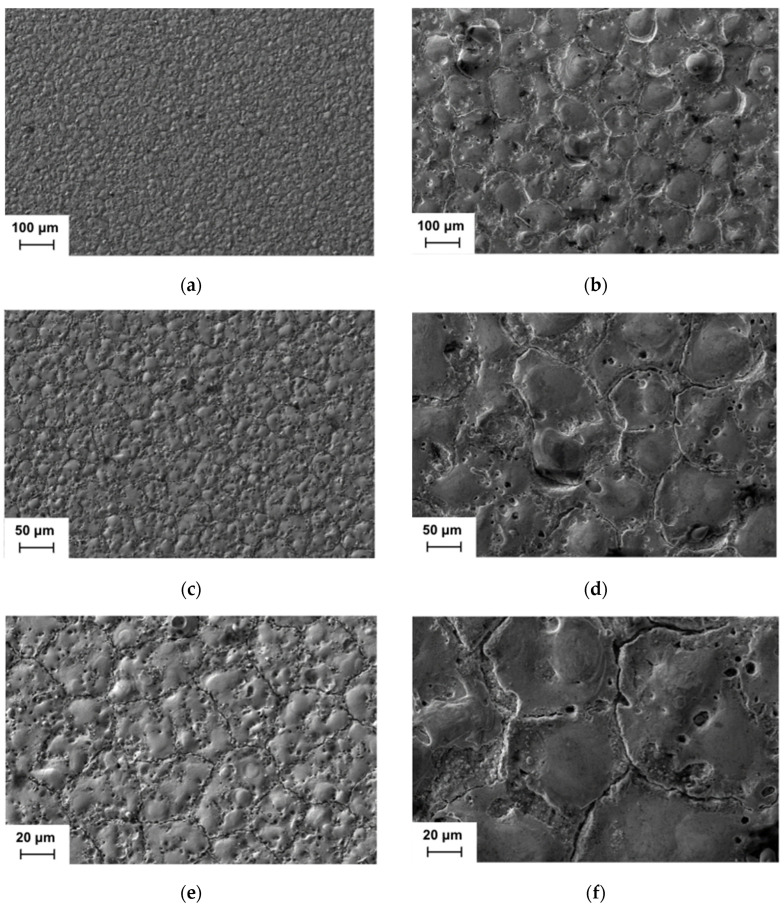
SEM micrographs for the EDM’ed WC: (**a**) minimum Ra case at 250×; (**b**) maximum Ra case at 250x; (**c**) minimum Ra case at 500×; (**d**) maximum Ra case at 500×; (**e**) minimum Ra case at 1000×; (**f**) maximum Ra case at 1000×.

**Figure 8:**
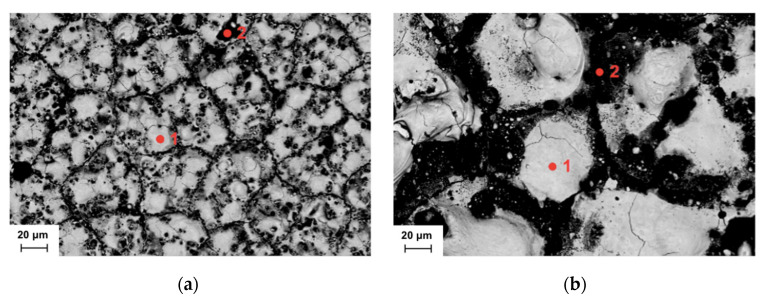
Locations where the EDX spectra are to be taken: (**a**) minimum Ra case at 1000×; (**b**) maximum Ra case at 1000×.

The two study cases that were selected in order to compare their surface quality and the integrity of their corresponding EDM’ed surfaces were the experiments with the lowest and the highest Ra values, which turned out to be E1 and E12, respectively. E1 (with a minimum Ra = 0.47 µm) was the experiment with I = 2 A and t_i_ = 10 µs, which had the lowest values for both the intensity and pulse time. On the contrary, E12 (with a maximum Ra = 2.53 µm) was the experiment which had the highest values for these two parameters, that is to say, I = 6 A and t_i_ = 100 µs. All of the SEM micrographs shown in Figure 7 were taken at different magnifications (250×, 500×, and 1000×) with a SEM microscope and using secondary electrons. As can be observed from Figure 7, the amount of resolidified material and the size of both the EDM craters and the pores are clearly greater in the case of the maximum Ra (see Figure 7b,d,f) in comparison with the case of the minimum Ra (see Figure 7a,c,e). Moreover, the length of the cracks seems to be clearly higher in the case of the maximum Ra, as can be seen in Figure 8. This is in line with previous research, which has demonstrated that higher energy levels—primarily determined by the current intensity—result in a greater surface density and increased damage [33,34]. To mitigate the risk of excessive damage to the heat-affected layers of the machined WC and WC-Co, a similar approach to minimizing the Ra and Rt should be employed, since the current intensity and pulse time have been identified as the two key electrical machining parameters which have the most influence on this process [35,36]. In practice, it is common to employ a combination of machining stages. Initial roughing phases are characterized by a high MRR but result in significant surface damage. These are followed by a subsequent finishing phase, which utilizes a lower current intensity and pulse time to refine the surface and make it suitable for industrial applications.

**Figure 9:**
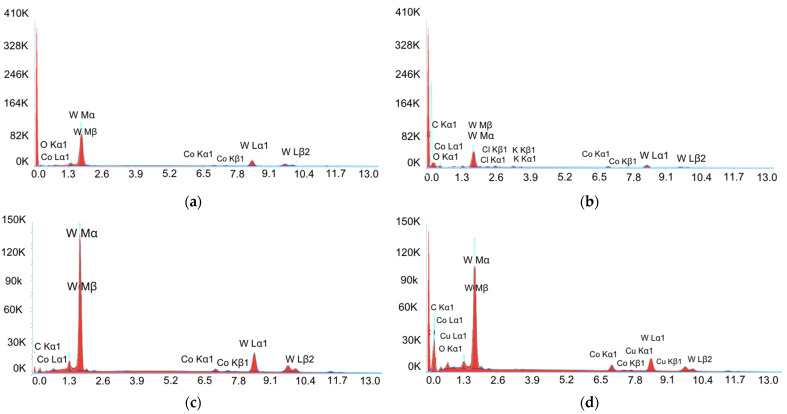
EDX spectra obtained for the locations shown in Figure 2: (**a**) spot “1” for the minimum Ra case; (**b**) spot “2” for the minimum Ra case; (**c**) spot “1” for the maximum Ra case; (**d**) spot “2” for the maximum Ra case.

Figure 8a,b show the SEM micrographs corresponding to those in Figure 7e,f but taken with backscattered electrons. The following two different types of zones may be clearly appreciated: one which is brighter (the spots marked with “1”) and another which is darker (the spots marked with “2”). Furthermore, it may be pointed out that darker zones are much more porous than brighter zones. In order to verify if there was any difference from the point of view of their chemical content, the EDX spectra (see Figure 9) were obtained at these two zones for both cases (the minimum Ra and maximum Ra).

With respect to the minimum Ra case, from the EDX analyses shown in Figure 9a,b, the content of cobalt is 1.66% (weight percentage) for spot “1” and 4.53% for spot “2”. Furthermore, the content of tungsten is 96.62% for spot “1” and 53.05% for spot “2”. As a consequence, it may be stated that bright zones are much richer in tungsten content than dark zones, but poorer in cobalt. This may be linked to the fact that dark zones (richer in cobalt, which is the metallic phase) are also more porous, as the material is more easily removed by the EDM process at these zones because of their higher value of electrical conductivity in relation to the ceramic phase, which is more present at the bright zones. Similarly, in the case of the maximum Ra, the EDX analyses (see Figure 9c,d) allows for this same conclusion to be reached, since the cobalt content is 2.45% for spot “1” and 4.32% for spot “2”, that is, higher in the dark zones (which are more attacked) rather than the bright zones (less attacked). On the contrary, the tungsten content is 88.63% for spot “1” and 48.49% for spot “2”. Moreover, the presence of copper content, which can be observed in the EDX spectrum from Figure 9d, may be attributed to the use of copper electrodes for the EDM process.

**Figure 10:**
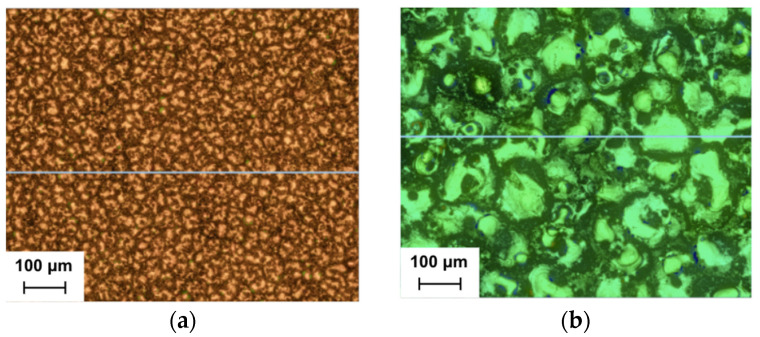
Depth maps for (**a**) the minimum Ra case; (**b**) the maximum Ra case.

**Figure 11:**
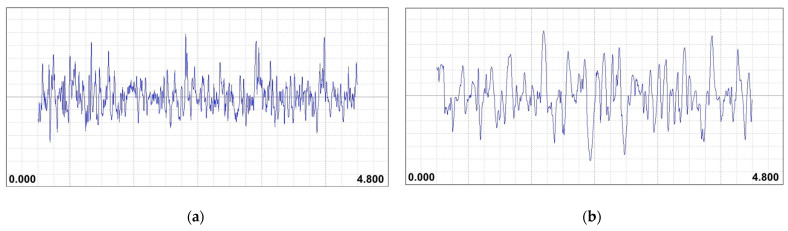
Roughness profile examples for (**a**) the minimum Ra case; (**b**) the maximum Ra case. The evaluation length is 4 mm for each profile. Each subdivision equates to 0.200 mm.

Additionally, as illustrated in Figure 10a,b, the software of the utilized confocal microscope generated a depth map for both E1 and E12, which allowed for another clear visual comparison of their topographical differences. The more energetic conditions of the maximum case resulted in larger craters, attributable to the increased volume of the molten material, which led to a coarser surface finish. This is comparable to the results observed in the roughness profiles generated by the profile rugosimeter for both cases (see Figure 11a,b). In the minimum case, the profile exhibits many more peaks and valleys of a smaller magnitude, while the maximum case shows fewer irregularities but with larger dimensions.

### 3.2. Analisys of Material Removal Rate (MRR)

The analysis was reiterated with a focus on the MRR as the primary system response. This focus is particularly significant, as it provides crucial insights into the efficacy and speed of the EDM process for WC. Equation (5) provides the mathematical representation of the model, which achieved an R^2^ of 98.77%, as follows:

(5) $$MRR=-0.469645+0.292465\cdot I+0.000776222\cdot t_{i}-0.0137781\cdot I^{2}\;-0.000014425\cdot I\cdot t_{i}-0.0000147222\cdot t_{i}^{2}$$

**Figure 12:**
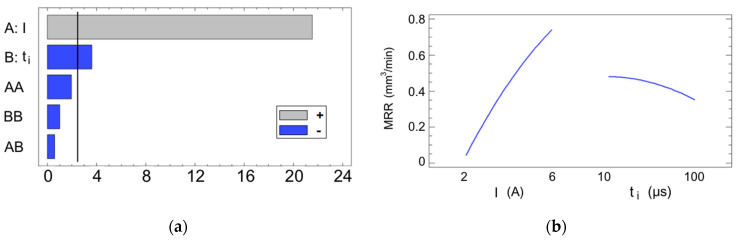
(**a**) Pareto chart for the MRR; (**b**) main effects plot for the MRR.

The highest MRR value achieved in this study was 0.7927 mm^3^/min, which corresponded to E6 with I = 6 A and t_i_ = 40 µs. In this case, the analysis of variance (see Table 3) confirmed that the discharge current is by far the most significant factor, followed distantly by the pulse time. This is clearly illustrated in the Pareto chart (see Figure 12a), where the current intensity exhibits a strongly positive effect on the machining speed. As previously justified for the SR, the MRR is also inherently related to the energy transfer of the process and the consequent rate of material melting and vaporization, thus the main influence of I. It may be noted that, in contrast to the SR, an increase in t_i_ does not exert a positive influence on the MRR, which seems counter-intuitive, since longer pulse times are related to higher energy levels. The results showed that, even though the pulse time is deemed relevant, increasing it has a relatively minimal impact on the machining speed, with the exception of a slight reduction when approaching the highest level of 100 μs (see Figure 12b). This effect has been noted in other studies for WC, as well as in other materials [7,37,38,39]. In his study about the energy transfer on WC samples, Singh [40] discovered that increasing the pulse time to around the 100 µs mark for different current levels led to a slowing down and an ultimate decrease in the MRR due to less primary energy fraction being transferred to the workpiece.

**Figure 13:**
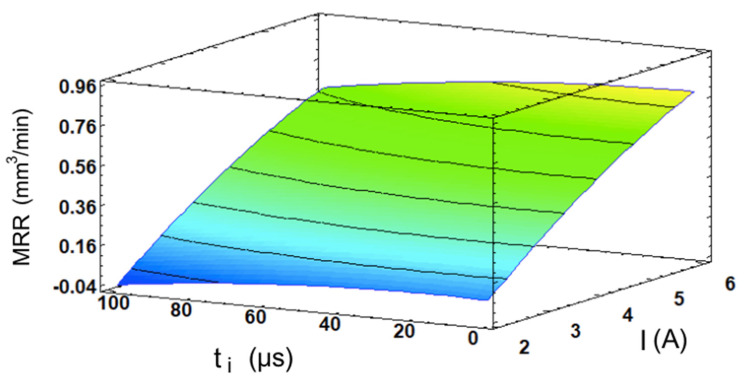
The I- and t_i_-dependent response surface for the MRR.

No statistically significant interaction effects were observed in the analysis. Combined with the dominant influence of the current intensity on the MRR, the response surface in Figure 13 exhibits a nearly planar geometry, with its gradient oriented primarily in the direction of the current intensity.

### 3.3. Analisys of Electrode Wear (EW)

Finally, a final statistical analysis was conducted on the electrode wear, which measures the percentage of the electrode material lost relative to the material removed. In industry, it is often critical to reduce wear to extend the tool life and to maintain dimensional tolerances. Prior to the analysis, it is important to note that, for E7 and E10, the electrode wear exceeded 100%, where this indicates that more electrode material was consumed than the volume of workpiece material removed during machining. This outcome is undesirable, as it suggests excessive electrode wear and, in both cases, the obtained MRR was extremely low. This time, the proposed model was calculated with an R^2^ = 88.16% and is defined by Equation (6). The quality of the fit was somewhat lower compared to the other cases, likely due to the greater numerical dispersion in the experimental results.

(6) $$EW=166.186-101.841\cdot I+2.43838\cdot t_{i}+14.1356\cdot I^{2}\;-0.735733\cdot I\cdot t_{i}+0.0133639\cdot t_{i}^{2}$$

**Figure 14:**
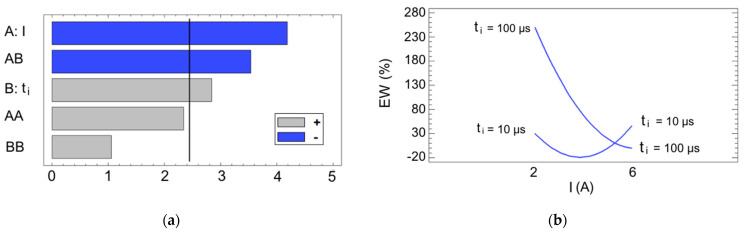
(**a**) Pareto chart for the EW; (**b**) interaction diagram for the EW.

Once again, the analysis of variance presented in Table 4 demonstrates that the current intensity is the most significant factor, closely followed by the interaction between the intensity and the pulse time, which exhibits a comparable level of importance to the primary factor, as shown in Figure 14a. The nature of this interaction is illustrated in the interaction diagram from Figure 14b, where it is evident that the choice of pulse time is critical at low intensity levels. In these cases, it may cause the EW to spike from acceptable levels to over 200%. In contrast, at a current intensity level of 6 A, the pulse time becomes less relevant and the EW stabilizes at lower and more consistent values, around 10%, as was previously shown in Table 1.

**Figure 15:**
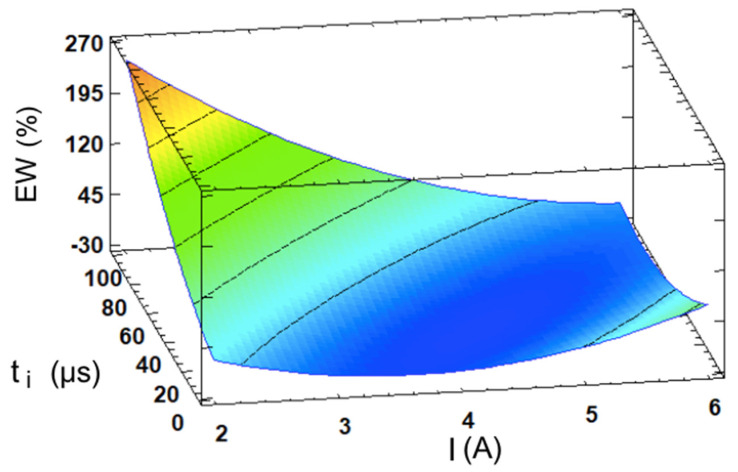
The I- and t_i_-dependent response surface for the EW.

Figure 15 presents the estimated response surface for this case. It may be highlighted that there exists a critical region of high wear, as well as a valley of optimal values which represents a strategy for minimizing tool wear. This optimal region is observed at intermediate levels of both design parameters. EDM roughing stages, which correspond to a higher current intensity, usually cause less electrode wear compared to finishing stages [12]. In general, it is thought that longer spark times lead to a decrease in the wear due to a protective molten material layer which appears on the electrode surface caused by larger plasma channels [25], and this is true for materials such as steel [41]. Nevertheless, this is not the case for WC-Co, since a high wear spike occurs precisely at high pulse times due to its very positive influence, where this effect has also been observed for WC in other research studies [7,42].

## 4. Conclusions

In this present study, an experimental analysis was conducted to determine the influence of the machining parameters of the current (I) and pulse time (t_i_) on the die-sinking EDM process of cobalt-bonded tungsten carbide (94WC-6Co), given the large number of engineering applications that this hard-to-machine material presents. Its machinability was studied in terms of the surface roughness (Ra and Rt), material removal rate (MRR), and electrode wear (EW). The surface characteristics of the minimum and maximum Ra cases were also examined using scanning electron microscopy. Considering that the work intervals for I and t_i_ were from 2 A to 6 A, and from 10 μs to 100 μs, respectively, the following conclusions may be drawn:

- In all four of the studied output parameters (Ra, Rt, MMR, and EW), the most influential factor in the EDM process was the current intensity, whereas the pulse time showed smaller but still significant influence.
- To achieve improved surface finishes, reflected by low Ra and Rt values, the process should be set to operate at a low energy level. This requires using a low current intensity and short pulse duration, which reduces the abrupt melting of the surface material and results in smaller and shallower craters on the EDM’ed surface. The minimum Ra and Rt values obtained were 0.47 μm and 4.49 μm (I = 2 A; t_i_ = 10 μs), and the maximum values were 2.53 μm and 19.90 μm (I = 6 A; t_i_ = 100 μs).
- Surface analysis revealed insights into the topography and composition of the previously mentioned minimum and maximum cases. Significant dimensional differences of the craters were observed, which correspond to the obtained roughness values. Additionally, two distinct regions were identified based on their chemical compositions, where the two of them showed a highly heterogeneous distribution of both tungsten and cobalt, which varied in selected spots for the minimum Ra case from 96.62% to 53.05%, and from 1.66% to 4.56%, respectively. Areas with a higher cobalt content experienced greater melting due to an increased electrical conductivity.
- For the MRR, the current intensity was the most significant factor, with a much greater influence than the pulse time. To obtain a higher MRR, a high current intensity and low pulse time should be utilized. This is partially consistent with the case of the surface finish, as more energetic pulses caused by a high current intensity are known to enhance the removal rate. However, the pulse time has been reported to show a different influence depending on the machined material, which has been repeatedly proven to be negative in the case of WC–Co. The minimum MRR value obtained was 0.0043 mm^3^/min (I = 2 A; t_i_ = 100 μs) and the maximum value was 0.7927 mm^3^/min (I = 6 A; t_i_ = 40 μs).
- Regarding the EW, there were instances where the EDM process occurred in reverse, where this means that more material was removed from the electrode than from the workpiece, which is undesirable. The maximum EW value was 297.34% and occurred at I = 2 A and t_i_ = 100 μs. Oppositely, the best results (9.51% at I = 6 A and t_i_ = 40 μs) were obtained by maintaining medium levels of both factors, as a strong interaction effect between them was observed, which was almost as significant as the current intensity.

This study offers valuable insights into the effects of the current intensity and pulse time on the EDM process for a specific grade of WC-Co, while key directions for future research work have also been identified. These include exploring the influence of different WC-Co grades by varying the physical properties, such as the thermal conductivity, and conducting detailed studies of the electrode wear morphology. Additionally, employing advanced statistical methods or machine learning approaches could help to capture higher-order effects and interactions beyond the primary electrical parameters in order to develop deeper and more dynamic models. These efforts to be made in future research work will extend the findings of this present study and enhance the understanding of EDM processes across diverse materials and conditions.

## Figures and Tables

**Table 1:** Summary of the SR, MRR, and EW results.

Exp. No.	Input Variables		Output Variables			
	I (A)	t_i_ (μs)	Ra (μm)	Rt (μm)	MRR (mm^3^/min)	EW (%)
1	2	10	0.47	4.49	0.0696	24.64
2	4	10	1.10	9.41	0.5120	13.85
3	6	10	1.48	11.92	0.7372	13.57
4	2	40	0.58	6.12	0.0200	66.90
5	4	40	1.62	14.31	0.5051	9.88
6	6	40	1.89	15.58	0.7927	9.51
7	2	70	0.58	5.35	0.0097	121.87
8	4	70	1.95	16.20	0.3779	12.07
9	6	70	2.38	17.87	0.7314	9.99
10	2	100	0.61	4.76	0.0043	297.34
11	4	100	2.20	17.03	0.3235	15.01
12	6	100	2.53	19.90	0.6312	10.14

**Table 2:** Analysis of variance (ANOVA) for the Ra parameter output.

Source	Sum of Squares	df	Mean Square	f-Value	*p*-Value
A:I	4.5421	1	4.5421	278.50	0.0000
B:t_i_	0.983552	1	0.983552	60.31	0.0002
AA	0.430944	1	0.430944	26.42	0.0021
AB	0.256	1	0.256	15.70	0.0074
BB	0.0314163	1	0.0314163	1.93	0.2145
Error	0.0978546	6	0.0163091		
Total	6.34187	11			

**Table 3:** Analysis of variance (ANOVA) for the MRR output.

Source	Sum of Squares	df	Mean Square	f-Value	*p*-Value
A:I	0.972245	1	0.972245	464.25	0.0000
B:t_i_	0.0272299	1	0.0272299	13.00	0.0113
AA	0.0080997	1	0.0080997	3.87	0.0968
AB	0.00074909	1	0.00074909	0.36	0.5717
BB	0.00210675	1	0.00210675	1.01	0.3546
Error	0.0125653	6	0.00209422		
Total	1.023	11			

**Table 4:** Analysis of variance (ANOVA) for the EW output.

Source	Sum of Squares	df	Mean Square	f-Value	*p*-Value
A:I	27,324.2	1	27,324.2	17.54	0.0058
B:t_i_	12584	1	12584	8.08	0.0295
AA	8525.48	1	8525.48	5.47	0.0579
AB	19,486.9	1	19,486.9	12.52	0.0123
BB	1735.93	1	1735.93	1.11	0.3318
Error	9348.31	6	1558.05		
Total	79,004.8	11			

## Data Availability

The original contributions presented in this study are included in the article material. Further inquiries can be directed to the corresponding authors.
